# Bis(2-phenyl­biguanidium) adipate tetra­hydrate

**DOI:** 10.1107/S1600536810049925

**Published:** 2010-12-11

**Authors:** Irena Matulková, Ivana Císařová, Ivan Němec

**Affiliations:** a Department of Inorganic Chemistry, Faculty of Science, Charles University in Prague, Hlavova 2030, 128 40 Prague 2, Czech Republic; b Department of Spectroscopy, J. Heyrovský Institute of Physical Chemistry of the ASCR, v.v.i., Dolejškova 3, 182 23 Prague 8, Czech Republic

## Abstract

In the title salt, 2C_8_H_12_N_5_^+^·C_6_H_8_O_4_^2−^·4H_2_O, the anion is located on a centre of symmetry. The observed supra­molecular network of the crystal structure is produced by ten different hydrogen bonds of the N—H⋯N, N—H⋯O and O—H⋯O types. One additional O—H group is not connected to an acceptor site.

## Related literature

For uses of biguanide complexes in medicine, see: Sirtori & Pasik (1994 ▶); Clement & Girreser (1999 ▶); Thompson *et al.* (1999 ▶); Ross *et al.* (2004 ▶); Woo *et al.* (1999 ▶); Watkins *et al.* (1987 ▶); Morain *et al.* (1994 ▶); Marchi *et al.* (1999 ▶); Shapiro *et al.* (1959*a* ▶,*b* ▶). The salts of biguanidium (1+) or (2+) cations have been tested for non-linear optical properties, see: Matulková *et al.* (2008 ▶, 2010 ▶); Martin *et al.* (1996 ▶); Martin & Pinkerton (1996 ▶); Pinkerton *et al.* (1978 ▶).
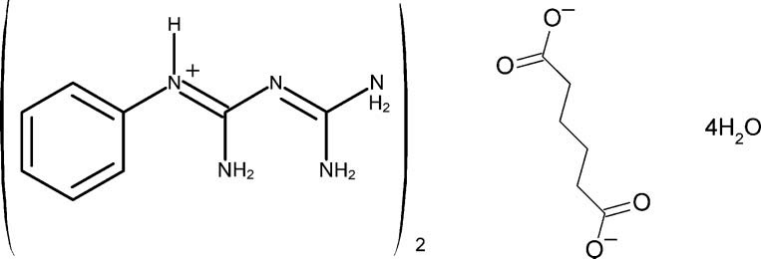


## Experimental

### 

#### Crystal data

2C_8_H_12_N_5_^+^·C_6_H_8_O_4_^2−^·4H_2_O
*M* *_r_* = 572.64
Triclinic, 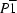

*a* = 7.1560 (1) Å
*b* = 10.8670 (2) Å
*c* = 11.1410 (2) Å
α = 61.5590 (9)°
β = 88.682 (1)°
γ = 71.702 (1)°
*V* = 714.93 (2) Å^3^
*Z* = 1
Mo *K*α radiation
μ = 0.10 mm^−1^
*T* = 150 K
0.4 × 0.4 × 0.3 mm

#### Data collection

Nonius KappaCCD area-detector diffractometer
20455 measured reflections
3260 independent reflections
2953 reflections with *I* > 2σ(*I*)
*R*_int_ = 0.025

#### Refinement

*R*[*F* ^2^ > 2σ(*F* ^2^)] = 0.035
*wR*(*F* ^2^) = 0.093
*S* = 1.06
3260 reflections
181 parameters
H-atom parameters constrained
Δρ_max_ = 0.25 e Å^−3^
Δρ_min_ = −0.25 e Å^−3^

### 

Data collection: *COLLECT* (Hooft, 1998 ▶) and *DENZO* (Otwinowski & Minor, 1997 ▶); cell refinement: *COLLECT* and *DENZO*; data reduction: *COLLECT* and *DENZO*; program(s) used to solve structure: *SIR92* (Altomare *et al.*, 1994 ▶); program(s) used to refine structure: *SHELXL97* (Sheldrick, 2008 ▶); molecular graphics: *PLATON* (Spek, 2009 ▶); software used to prepare material for publication: *SHELXL97*.

## Supplementary Material

Crystal structure: contains datablocks global, I. DOI: 10.1107/S1600536810049925/im2248sup1.cif

Structure factors: contains datablocks I. DOI: 10.1107/S1600536810049925/im2248Isup2.hkl

Additional supplementary materials:  crystallographic information; 3D view; checkCIF report

## Figures and Tables

**Table 1:** Hydrogen-bond geometry (Å, °)

*D*—H⋯*A*	*D*—H	H⋯*A*	*D*⋯*A*	*D*—H⋯*A*
N5—H1⋯O1	0.89	2.12	2.982 (1)	163
N2—H2*A*⋯O2^i^	0.94	1.92	2.837 (1)	168
N2—H2*B*⋯O2^ii^	0.88	2.07	2.855 (1)	149
N4—H4*A*⋯N3^iii^	0.90	2.14	3.041 (1)	178
N4—H4*B*⋯O1*W*	0.92	2.23	2.998 (1)	141
N1—H5*A*⋯O1^i^	0.94	1.95	2.882 (1)	171
N5—H5*B*⋯O1*W*	0.92	2.03	2.897 (1)	158
O1*W*—H11⋯O1^iv^	0.85	1.99	2.825 (1)	168
O1*W*—H12⋯O2*W*^v^	0.92	1.86	2.781 (1)	174
O2*W*—H22⋯O2	0.93	1.89	2.797 (1)	165

Symmetry codes: (i) 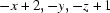; (ii) 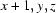; (iii) 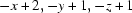; (iv) 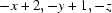; (v) 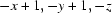.

**Fig. 1.**
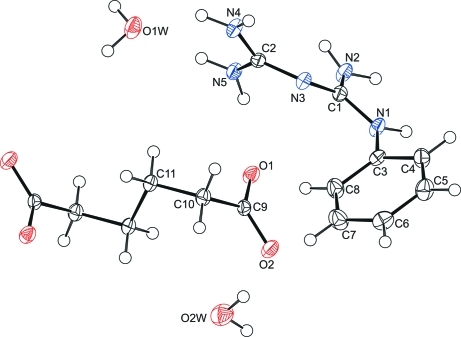
Atom-labelling scheme of N-phenylbiguanidium(1+) adipate dihydrate. Displacement ellipsoids are drawn at the 50% probability level.

**Fig. 2.**
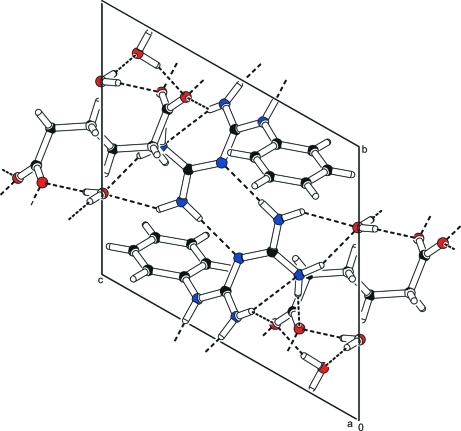
Packing scheme of the crystalstructure of N-phenylbiguanidium(1+) adipate dihydrate. Hydrogen bonds are indicated by dashed lines.

## supplementary crystallographic information

### Comment

The wide area of application of biguanides involves the field of medical research (Sirtori & Pasik, 1994; Clement & Girreser, 1999), especially in the treatment of diabetes mellitus (*N,N*-dimethylbiguanide and *N*-phenylethylbiguanide) (Thompson *et al.*, 1999; Ross *et al.*, 2004; Woo *et al.*, 1999). Biguanide derivatives are also used in the synthesis of antimalarial drugs (*N*-butylbiguanide) (Watkins *et al.*, 1987), and in therapeutic treatment of pain, anxiety, memory disorders (Morain *et al.*, 1994) and hypoglycaemic activity (Marchi *et al.*, 1999; Sirtori & Pasik, 1994; Shapiro *et al.*, 1959*a*, 1959*b*).

All the ionic crystal structures containing biguanide moieties are formed by relatively strong hydrogen bonds (Matulková *et al.*, 2010; Matulková *et al.*, 2008). These intermolecular and intramolecular hydrogen bonds can substantially affect the geometry of the biguanidium cations (Martin *et al.*, 1996; Martin & Pinkerton, 1996; Pinkerton *et al.*, 1978). It was found in general that, as the value of the angle between the planes (formed by three nitrogen atoms and one carbon atom) in the biguanide molecule increases, the bonding distances increase and thus the electron distribution changes.

The molecular conformation of the constituents of the title compound is illustrated in Fig. 1. Hydrogen bonds in the crystal structure are formed between *N*-phenylbiguanidium cations and adipate anions, between *N*-phenylbiguanidium cations and water molecules, as well as between the carboxylate functions and water molecules. Hydrogen-bonding geometries in the title compound are listed in Table 1 and are illustrated in Fig. 2. Additionally, two *N*-phenylbiguanidium cations form a dimer *via* two centrosymmetrically related N—H···N hydrogen bonds. These dimers are interconnected by adipate anions into a network further supported by hydrogen bonds involving water molecules. The lengths of the N—H···O hydrogen bonds range from 2.837 (1) to 3.000 (1) Å. O - H···O hydrogen bonds connect water molecules with adipate anions and range from 2.781 (1) to 2.825 (1) Å.

### Experimental

Crystals of the title compound were obtained from a solution of 0.3 g of *N*-phenylbiguanide (98%, Aldrich) and 0.53 g of adipic acid (purum, Lachema) in 4 ml of water and 8 ml of methanol. The solution was left to crystallize at room temperature for several weeks. The colourless crystals obtained were filtered off, washed with methanol and dried in a vacuum desiccator over KOH (m.p. 350-352 K).

Infrared spectra were recorded at room temperature using DRIFTS and nujol or fluorolube mull techniques on a Nicolet Magna 760 FTIR spectrometer with 2 cm^-1^ resolution (4 cm^-1^ resolution in far IR region) and Happ-Genzel apodization in the 85–4000 cm^-1^ region.

FTIR spectrum (cm^-1^): 3623 w; 3377 m; 3307 m; 3139 m; 3072 m; 2923 m; 2723 m; 1650 s; 1632 s; 1600 m; 1582 m; 1555 m; 1494 vw; 1456 vw; 1409 s; 1365 sh; 1317 m; 1303 s; 1290 s; 1266 s; 1200 m; 1169 w; 1153 vw; 1139 w; 1122 w; 1073 w; 1065 w; 1026 vw; 1003 vw; 936 w; 926 w; 911 w; 860 vw; 835 vw; 799 vw; 772 w; 741 m; 722 m; 714 vw; 696 m; 673 vw; 640 vw; 623 vw; 581 wb; 541 vw; 497 w; 483 w; 389 w; 316 w; 280 w; 235 w; 179 w.

Raman spectra of polycrystalline samples were recorded at room temperature on a Nicolet Magna 760 FTIR spectrometer equipped with the Nicolet Nexus FT Raman module (2 cm^-1^ resolution, Happ-Genzel apodization, 1064 nm Nd:YVO4 laser excitation, 450 mW power at the sample) in the 100–3700 cm^-1^ region.

FT Raman spectrum (cm^-1^): 3296 vw; 3196 wb; 3069 m; 3057 m; 2967 w; 2931 m; 2919 m; 2900 w; 2875 w; 1696 vw; 1647 vwb; 1601 s; 1586 m; 1545 w; 1515 w; 1494 m; 1444 w; 1424 m; 1411 m; 1362 vw; 1321 m; 1308 w; 1288 w; 1269 s; 1243 sh; 1229 sh; 1176 m; 1155 m; 1084 m; 1060 m; 1030 m; 1018 vw; 1003 vs; 991 vw; 963 vw; 936 m; 909 m; 885 m; 857 m; 775 w; 740 m; 728 w; 671 w; 638 w; 615 m; 539 m; 493 m; 482 w; 408 m; 388 m; 376 w; 274 m; 234 m; 183 m; 151 mb.

### Refinement

H atoms attached to C atoms were calculated in geometrically idealized positions, with C*sp*^3^ - H = 0.97 Å and C*sp*^2^ - H = 0.93 Å. The positions of H atoms attached to O and N atoms were localized on difference Fourier maps. All hydrogen atoms were constrained to ride on their parent atoms during refinement, with *U*_iso_(H) = 1.2 *U*_eq_(pivot atom).

### Crystal data

**Table:**

2C_8_H_12_N_5_^+^·C_6_H_8_O_4_^2^^−^·4H_2_O	*Z* = 1
*M**_r_* = 572.64	*F*(000) = 306
Triclinic, *P*1	*D*_x_ = 1.330 Mg m^−^^3^
Hall symbol: -P 1	Mo *K*α radiation, λ = 0.71073 Å
*a* = 7.1560 (1) Å	Cell parameters from 3257 reflections
*b* = 10.8670 (2) Å	θ = 1.0–27.5°
*c* = 11.1410 (2) Å	µ = 0.10 mm^−^^1^
α = 61.5590 (9)°	*T* = 150 K
β = 88.682 (1)°	Prism, colourless
γ = 71.702 (1)°	0.4 × 0.4 × 0.3 mm
*V* = 714.93 (2) Å^3^	

### Data collection

**Table:**

Nonius KappaCCD area-detector diffractometer	2953 reflections with *I* > 2σ(*I*)
Radiation source: fine-focus sealed tube	*R*_int_ = 0.025
graphite	θ_max_ = 27.4°, θ_min_ = 2.1°
Detector resolution: 9.091 pixels mm^-1^	*h* = −9→9
ω and π scans to fill the Ewald sphere	*k* = −14→14
20455 measured reflections	*l* = −14→14
3260 independent reflections	

### Refinement

**Table:**

Refinement on *F*^2^	0 restraints
Least-squares matrix: full	H-atom parameters constrained
*R*[*F*^2^ > 2σ(*F*^2^)] = 0.035	*w* = 1/[σ^2^(*F*_o_^2^) + (0.0427*P*)^2^ + 0.2629*P*] where *P* = (*F*_o_^2^ + 2*F*_c_^2^)/3
*wR*(*F*^2^) = 0.093	(Δ/σ)_max_ < 0.001
*S* = 1.06	Δρ_max_ = 0.25 e Å^−^^3^
3260 reflections	Δρ_min_ = −0.25 e Å^−^^3^
181 parameters	

### Special details

**Table:**

Geometry. All e.s.d.'s (except the e.s.d. in the dihedral angle between two l.s. planes) are estimated using the full covariance matrix. The cell e.s.d.'s are taken into account individually in the estimation of e.s.d.'s in distances, angles and torsion angles; correlations between e.s.d.'s in cell parameters are only used when they are defined by crystal symmetry. An approximate (isotropic) treatment of cell e.s.d.'s is used for estimating e.s.d.'s involving l.s. planes.
Refinement. Refinement of *F*^2^ against ALL reflections. The weighted *R*-factor *wR* and goodness of fit *S* are based on *F*^2^, conventional *R*-factors *R* are based on *F*, with *F* set to zero for negative *F*^2^. The threshold expression of *F*^2^ > σ(*F*^2^) is used only for calculating *R*-factors(gt) *etc*. and is not relevant to the choice of reflections for refinement. *R*-factors based on *F*^2^ are statistically about twice as large as those based on *F*, and *R*- factors based on ALL data will be even larger.

### Fractional atomic coordinates and isotropic or equivalent isotropic displacement parameters (Å^2^)

**Table:**

	*x*	*y*	*z*	*U*_iso_*/*U*_eq_	
C1	1.08265 (15)	0.19532 (11)	0.51868 (10)	0.0166 (2)	
C2	1.02860 (14)	0.43683 (11)	0.33714 (10)	0.0157 (2)	
N1	1.01068 (14)	0.10880 (10)	0.62783 (9)	0.0198 (2)	
H1	0.9966	0.3356	0.2376	0.024*	
N2	1.23411 (14)	0.12406 (10)	0.47705 (10)	0.0211 (2)	
H2A	1.2927	0.0211	0.5352	0.025*	
H2B	1.3012	0.1750	0.4191	0.025*	
N3	1.00775 (13)	0.34344 (10)	0.46586 (9)	0.01745 (19)	
N4	1.03596 (14)	0.56792 (10)	0.31118 (9)	0.0205 (2)	
H4A	1.0268	0.5929	0.3779	0.025*	
H4B	1.0357	0.6423	0.2236	0.025*	
N5	1.03464 (14)	0.40864 (10)	0.23239 (9)	0.01899 (19)	
H5A	1.0784	0.0048	0.6749	0.023*	
H5B	1.0304	0.4875	0.1479	0.023*	
C3	0.83808 (15)	0.16121 (11)	0.67987 (11)	0.0170 (2)	
C4	0.85199 (17)	0.11060 (13)	0.82069 (11)	0.0232 (2)	
H4	0.9739	0.0489	0.8776	0.028*	
C5	0.68398 (18)	0.15191 (14)	0.87700 (12)	0.0262 (2)	
H5	0.6938	0.1175	0.9715	0.031*	
C6	0.50251 (17)	0.24393 (13)	0.79302 (13)	0.0246 (2)	
H6	0.3900	0.2708	0.8308	0.030*	
C7	0.48929 (17)	0.29590 (14)	0.65196 (13)	0.0289 (3)	
H7	0.3678	0.3591	0.5950	0.035*	
C8	0.65620 (17)	0.25428 (14)	0.59510 (12)	0.0251 (2)	
H8	0.6461	0.2886	0.5006	0.030*	
C9	0.65021 (15)	0.26120 (11)	0.25517 (10)	0.0159 (2)	
C10	0.55128 (16)	0.42815 (11)	0.19755 (11)	0.0185 (2)	
H10A	0.6186	0.4595	0.2467	0.022*	
H10B	0.4142	0.4488	0.2143	0.022*	
C11	0.55403 (16)	0.51991 (11)	0.04279 (10)	0.0180 (2)	
H11A	0.4914	0.6250	0.0133	0.022*	
H11B	0.6910	0.5030	0.0260	0.022*	
O1	0.82344 (11)	0.21203 (8)	0.23165 (8)	0.02091 (18)	
O2	0.55463 (12)	0.17812 (8)	0.32638 (8)	0.02243 (18)	
O1W	0.98182 (13)	0.70284 (10)	0.00316 (8)	0.0287 (2)	
H11	1.0494	0.7325	−0.0619	0.034*	
H12	0.8552	0.7580	−0.0462	0.034*	
O2W	0.40159 (15)	0.11820 (11)	0.13910 (10)	0.0353 (2)	
H21	0.4200	0.0216	0.1821	0.042*	
H22	0.4504	0.1220	0.2138	0.042*	

### Atomic displacement parameters (Å^2^)

**Table:**

	*U*^11^	*U*^22^	*U*^33^	*U*^12^	*U*^13^	*U*^23^
C1	0.0182 (5)	0.0161 (5)	0.0150 (5)	−0.0064 (4)	0.0028 (4)	−0.0069 (4)
C2	0.0126 (4)	0.0157 (5)	0.0168 (5)	−0.0042 (4)	0.0033 (4)	−0.0070 (4)
N1	0.0214 (4)	0.0130 (4)	0.0193 (4)	−0.0042 (3)	0.0081 (4)	−0.0049 (3)
N2	0.0208 (5)	0.0142 (4)	0.0214 (4)	−0.0042 (3)	0.0091 (4)	−0.0050 (4)
N3	0.0211 (4)	0.0142 (4)	0.0158 (4)	−0.0063 (3)	0.0057 (3)	−0.0065 (3)
N4	0.0296 (5)	0.0158 (4)	0.0166 (4)	−0.0101 (4)	0.0067 (4)	−0.0071 (4)
N5	0.0246 (5)	0.0182 (4)	0.0151 (4)	−0.0103 (4)	0.0043 (3)	−0.0073 (3)
C3	0.0197 (5)	0.0140 (5)	0.0186 (5)	−0.0077 (4)	0.0064 (4)	−0.0080 (4)
C4	0.0222 (5)	0.0234 (5)	0.0182 (5)	−0.0051 (4)	0.0024 (4)	−0.0075 (4)
C5	0.0303 (6)	0.0293 (6)	0.0185 (5)	−0.0092 (5)	0.0081 (4)	−0.0124 (5)
C6	0.0229 (5)	0.0248 (6)	0.0299 (6)	−0.0083 (4)	0.0110 (5)	−0.0166 (5)
C7	0.0195 (6)	0.0327 (6)	0.0277 (6)	−0.0031 (5)	0.0016 (5)	−0.0133 (5)
C8	0.0236 (6)	0.0303 (6)	0.0171 (5)	−0.0069 (5)	0.0026 (4)	−0.0099 (5)
C9	0.0177 (5)	0.0159 (5)	0.0121 (4)	−0.0055 (4)	0.0011 (4)	−0.0055 (4)
C10	0.0194 (5)	0.0152 (5)	0.0176 (5)	−0.0041 (4)	0.0027 (4)	−0.0068 (4)
C11	0.0199 (5)	0.0132 (5)	0.0171 (5)	−0.0053 (4)	0.0013 (4)	−0.0047 (4)
O1	0.0181 (4)	0.0159 (4)	0.0228 (4)	−0.0045 (3)	0.0060 (3)	−0.0061 (3)
O2	0.0218 (4)	0.0176 (4)	0.0228 (4)	−0.0080 (3)	0.0078 (3)	−0.0056 (3)
O1W	0.0340 (5)	0.0277 (4)	0.0182 (4)	−0.0116 (4)	0.0084 (3)	−0.0063 (3)
O2W	0.0436 (5)	0.0304 (5)	0.0366 (5)	−0.0187 (4)	0.0049 (4)	−0.0164 (4)

### Geometric parameters (Å, °)

**Table:**

C1—N2	1.3347 (13)	C5—H5	0.9300
C1—N3	1.3397 (13)	C6—C7	1.3877 (17)
C1—N1	1.3469 (13)	C6—H6	0.9300
C2—N4	1.3303 (13)	C7—C8	1.3897 (16)
C2—N5	1.3370 (13)	C7—H7	0.9300
C2—N3	1.3445 (13)	C8—H8	0.9300
N1—C3	1.4233 (13)	C9—O2	1.2622 (13)
N1—H5A	0.9432	C9—O1	1.2623 (13)
N2—H2A	0.9353	C9—C10	1.5198 (14)
N2—H2B	0.8755	C10—C11	1.5299 (14)
N4—H4A	0.8999	C10—H10A	0.9700
N4—H4B	0.9233	C10—H10B	0.9700
N5—H1	0.8934	C11—C11^i^	1.527 (2)
N5—H5B	0.9158	C11—H11A	0.9700
C3—C4	1.3874 (15)	C11—H11B	0.9700
C3—C8	1.3888 (15)	O1W—H11	0.8499
C4—C5	1.3911 (16)	O1W—H12	0.9225
C4—H4	0.9300	O2W—H21	0.8864
C5—C6	1.3824 (17)	O2W—H22	0.9334
			
N2—C1—N3	124.93 (9)	C4—C5—H5	119.9
N2—C1—N1	116.20 (9)	C5—C6—C7	119.57 (10)
N3—C1—N1	118.73 (9)	C5—C6—H6	120.2
N4—C2—N5	117.95 (9)	C7—C6—H6	120.2
N4—C2—N3	117.89 (9)	C6—C7—C8	120.50 (11)
N5—C2—N3	124.12 (9)	C6—C7—H7	119.8
C1—N1—C3	125.27 (9)	C8—C7—H7	119.8
C1—N1—H5A	118.6	C3—C8—C7	119.77 (10)
C3—N1—H5A	116.1	C3—C8—H8	120.1
C1—N2—H2A	116.5	C7—C8—H8	120.1
C1—N2—H2B	118.5	O2—C9—O1	123.20 (9)
H2A—N2—H2B	120.5	O2—C9—C10	117.75 (9)
C1—N3—C2	121.23 (9)	O1—C9—C10	119.03 (9)
C2—N4—H4A	120.7	C9—C10—C11	113.44 (8)
C2—N4—H4B	123.1	C9—C10—H10A	108.9
H4A—N4—H4B	115.8	C11—C10—H10A	108.9
C2—N5—H1	121.2	C9—C10—H10B	108.9
C2—N5—H5B	114.6	C11—C10—H10B	108.9
H1—N5—H5B	119.8	H10A—C10—H10B	107.7
C4—C3—C8	119.78 (10)	C11^i^—C11—C10	112.27 (11)
C4—C3—N1	118.26 (10)	C11^i^—C11—H11A	109.2
C8—C3—N1	121.87 (10)	C10—C11—H11A	109.2
C3—C4—C5	120.12 (11)	C11^i^—C11—H11B	109.2
C3—C4—H4	119.9	C10—C11—H11B	109.2
C5—C4—H4	119.9	H11A—C11—H11B	107.9
C6—C5—C4	120.25 (11)	H11—O1W—H12	99.6
C6—C5—H5	119.9	H21—O2W—H22	97.8

Symmetry codes: (i) −*x*+1, −*y*+1, −*z*.

### Hydrogen-bond geometry (Å, °)

**Table:**

*D*—H···*A*	*D*—H	H···*A*	*D*···*A*	*D*—H···*A*
N5—H1···O1	0.89	2.12	2.982 (1)	163
N2—H2A···O2^ii^	0.94	1.92	2.837 (1)	168
N2—H2B···O2^iii^	0.88	2.07	2.855 (1)	149
N4—H4A···N3^iv^	0.90	2.14	3.041 (1)	178
N4—H4B···O1W	0.92	2.23	2.998 (1)	141
N1—H5A···O1^ii^	0.94	1.95	2.882 (1)	171
N5—H5B···O1W	0.92	2.03	2.897 (1)	158
O1W—H11···O1^v^	0.85	1.99	2.825 (1)	168
O1W—H12···O2W^i^	0.92	1.86	2.781 (1)	174
O2W—H22···O2	0.93	1.89	2.797 (1)	165

Symmetry codes: (ii) −*x*+2, −*y*, −*z*+1; (iii) *x*+1, *y*, *z*; (iv) −*x*+2, −*y*+1, −*z*+1; (v) −*x*+2, −*y*+1, −*z*; (i) −*x*+1, −*y*+1, −*z*.
